# Lactylated histone H3K18 as a potential biomarker for the diagnosis and prediction of the severity of pancreatic cancer

**DOI:** 10.1016/j.clinsp.2024.100544

**Published:** 2024-11-26

**Authors:** Jinping Hou, Mingsong Guo, Yongqiong Li, Yijin Liao

**Affiliations:** a Department of Hepatological Surgery, The Sixth People's Hospital of Chengdu, Chengdu, PR China; b Department of Emergency, The Second People's Hospital of Chengdu, Chengdu, PR China; c Chengdu Qinglong Community Health Service Center, Chengdu, PR China

**Keywords:** Pancreatic cancer, Histone, Lactylation, H3K18

## Abstract

**Background:**

Lactylation plays an essential role in pancreatic cancer, but the precise role of lactylated histone in the diagnosis and prognosis of pancreatic cancer remains to be further clarified.

**Methods:**

Twenty-one patients diagnosed with pancreatic cancer were enrolled in this study, and the clinicopathologic characteristics were collected. Lactylation levels of total proteins and histone H3 Lysine-18 (H3K18) of tissues were determined by western blotting and laboratory indicators including serum levels of lactate, Cancer Antigen 19–9 (CA19–9), and Carcinoembryogenic Antigen (CEA) were obtained.

**Results:**

Total protein lactylation was found in both pancreatic cancer tissues and para-carcinoma normal tissues, and was more potent in tumor tissues. H3K18la was also highly expressed tumor tissues. Furthermore, H3K18la protein expression correlated positively with serum lactate (*r* = 0.774, *p* < 0.001), CA19–9 (*r* = 0.744, *p* < 0.001), and CEA (*r* = 0.589, *p* < 0.01). The Area Under the Curve (AUC) of H3K18la for the diagnosis of pancreatic cancer was 0.848 in serum (*p* < 0.001).

**Conclusion:**

The present findings suggested that H3K18 may be used as a novel potential biomarker for the diagnosis and prognosis of pancreatic cancer patients.

## Highlights

• High level of Pan Lysine Lactylation (Pan-Kla) is found in pancreatic cancer tissues.
• H3K18la levels are increased in pancreatic cancer tissues.
• H3K18la is related to the severity and prognosis of pancreatic cancer.

## Introduction

Pancreatic cancer is a highly lethal form of cancer. The early symptoms of pancreatic cancer are not obvious, and it is easy to be misdiagnosed as pancreatitis and ulcers, so most patients with pancreatic cancer are already in the advanced stage of diagnosis.^1^^,^^2^ In the process of invasion and metastasis of pancreatic cancer, the changes in gene function and phenotype of tumor cells are directly reflected in the changes in pancreatic cancer protein level.^3^^,^^4^ The altered proteins play an important role in regulating the biological processes of cell cycle, differentiation, proliferation, metabolism, and apoptosis, which are closely related to the treatment and prognosis of pancreatic cancer patients.^5^^,^^6^

Studies on pancreatic cancer have shown that the abnormal expression and function of some oncogenes or tumor suppressor genes are caused by epigenetic modification.^7^^,^^8^ Histone is composed of H2A, H2B, H3, and H4 protein dimers in octagonal shape, and each component has a dynamic regulation effect on chromatin. Among them, histone H3 has been studied most deeply, and its 24 amino acids at the N-terminal can be modified by methylation, acetylation, ubiquitination and other behaviors to play different transcriptional regulatory roles.^9^^,^^10^ Histone methylation can inhibit or promote transcription, while acetylation usually promotes transcription. Recently, Lysine Lactylation (Kla) of histone is a newly reported epigenetic modification that activates gene transcription.^11^ Intriguingly, lactate, a product of glycolysis in tumors, stimulates histone lactylation and subsequently activates downstream gene expression.^11^ Thus, it is interesting to explore the potential function of histone lactylation in tumors. For instance, histone lactylation of YTHDF2 contributes to tumorigenesis of ocular melanoma.^12^ Suppression of H3 histone lactylation inhibits liver cancer progress.^13^ However, in pancreatic cancer, the study of histone lactylation in pancreatic cancer is still in its infancy.

Increased histone H3 Lysine-18 lactylation (H3K18la) levels have been reported in various diseases. For instance, elevation of H3K18la protein levels was associated with sepsis and septic shock.^14^ Therefore, the authors detected the level of the previously identified H3K18la in tissues of pancreatic cancer in this study to assess its role in pancreatic cancer. The underlying mechanisms and physiological relevance were further detected through comparisons of cancer markers that have been clinically validated from the same patients.

## Materials and methods

### Study design and participants

A total of 21 patients diagnosed with pancreatic cancer at The Sixth People's Hospital of Chengdu were included in this study. Patients with pancreatic cancer confirmed by histopathology (*n* = 13) or cytology (*n* = 8) were included in the study. After the blood supply, location, size and shape of the tumor were determined by computerized tomography, pathological tissue or shed cells were obtained by duodenoscopy, and pathological examination confirmed the lesion. All patients had not received adjuvant chemoradiation or immunotherapy before radical or palliative surgery and had not recently developed infection or autoimmune disease. Immunohistochemical staining was performed on the pathological tissues collected after surgery: specific antibodies were bound to proteins in the tissues to detect molecular markers on the surface of tumor cells. Common markers included CK7, CK20, CEA, and CA19–9. The boundary between cancerous tissue and normal pancreatic parenchyma was determined by immunohistochemical staining. Moreover, postoperative tissue samples (including cancer/group T and para-cancer tissue/group N) after radical surgery and peripheral venous blood samples (5‒10 mL) were collected from all patients. This study complied with the requirements of medical ethics of The Sixth People's Hospital of Chengdu. After enrolment, the following baseline information was collected: age, sex, and comorbidities. The following laboratory indicators based on the same collection date were also assessed: serum lactate, serum Cancer Antigen 19–9 (CA19–9), and Carcinoembryogenic Antigen (CEA) level.

### Protein extraction and western blot

Tissues were homogenized in a lysis buffer containing protease and phosphatase inhibitor (Sigma-Aldrich). The total extracted proteins were quantified by a BAC kit and loaded for SDS-PAGE for electrophoresis. Afterwards, proteins were transferred to PVDF membranes, and blocked by 10 % skim milk. Then membranes were incubated with primary antibodies including anti-l-Lactyl Lysine Rabbit pAb (PTM-1401, 1/500, PTM Biolabs) and anti-l-Lactyl-Histone H3 (Lys18) Rabbit mAb (PTM-1406RM, 1/500, PTM Biolabs) overnight at 4 °C followed by incubation with a secondary antibody. Immunoreactive bands were visualized using Western Blotting Luminal Reagent (Santa Cruz Biotech) according to the manufacturer's recommendation.

### Statistical analysis

IBM SPSS 22.0 software was used for all statistical analyses. Data were compared using Student's *t*-test or one-way analysis of variance, and the results are shown as the mean ± SD. Pearson correlation was applied. The diagnostic value was determined by Receiver Operating Characteristic (ROC) curve analysis; *p* < 0.05 was considered statistically significant.

## Results

### High level of pan lysine lactylation (Pan-Kla) is found in pancreatic cancer tissues

**Fig. 1:**
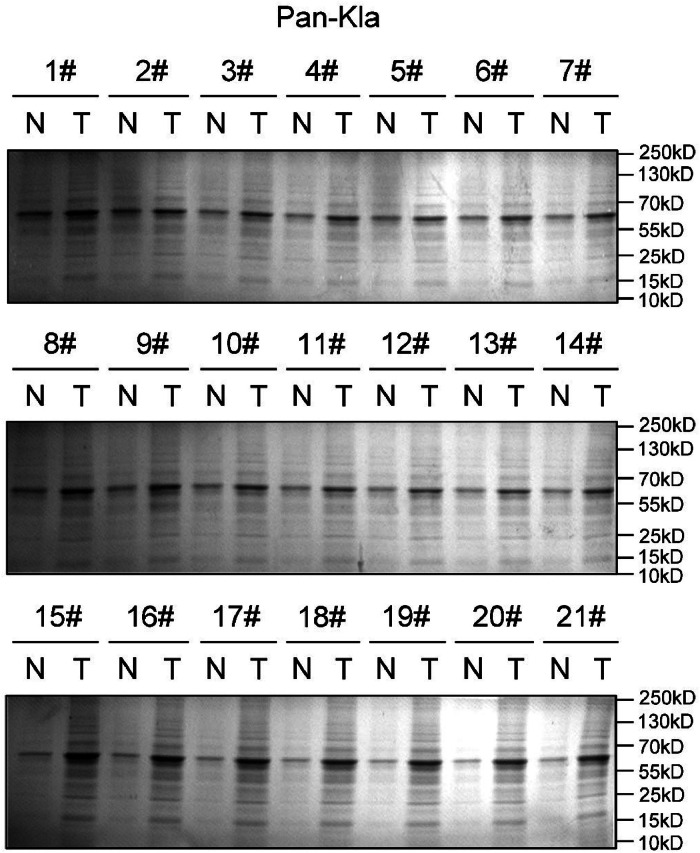
The pan lysine lactylation (Pan-Kla) levels in both pancreatic cancer tissues and para-carcinoma normal tissues collected from 21 patients.

A total of 21 patients with pancreatic cancer were tested in this study, and the Pan-Kla levels were detected in both pancreatic cancer tissues and para-carcinoma normal tissues. Lactylation was found in both normal and tumor tissues. Moreover, tumor tissues had higher levels of lactylation than normal tissues in the all-protein range (Fig. 1).

### H3K18la levels are increased in pancreatic cancer tissues

**Fig. 2:**
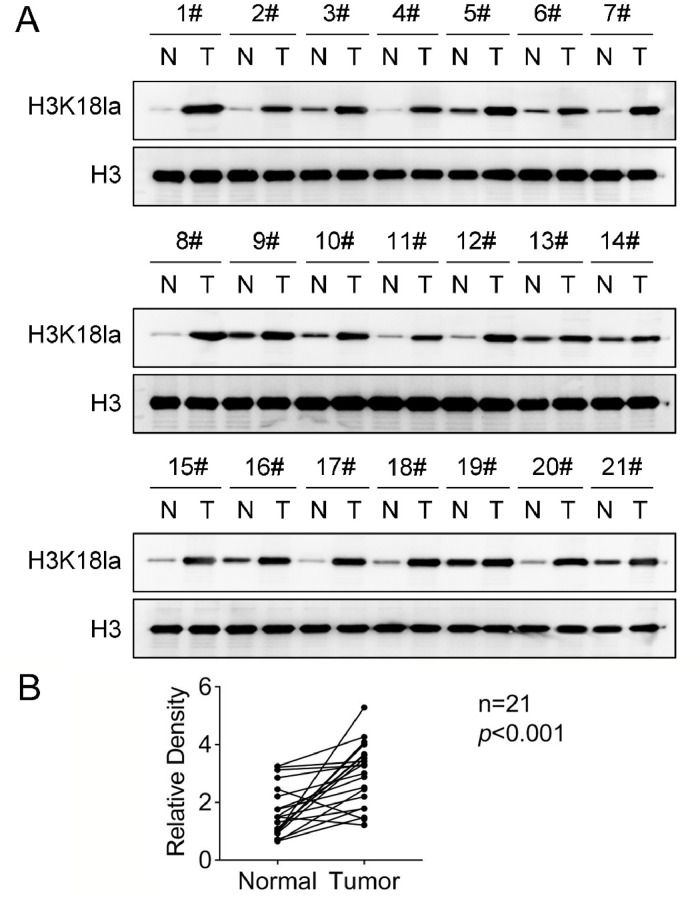
Expression of histone H3 lysine 18 lactylation (H3K18la) in pancreatic cancer tissues. (A) The representative protein brand of H3K18la, and (B) The relative density analysis.

H3K18la was expressed in both pancreatic cancer tissues and para-carcinoma normal tissues, and was significantly highly expressed in tumor tissues (Fig. 2A and B). The mean level of H3K18la relative density in patients was 2.979, and the relative density of H3K18la was <2.888 in 9 patients (Low group) and greater than 2.979 in 12 cases (High group). According to the clinicopathologic characteristics collected in Table 1, the authors found that high levels of H3K18la were significantly correlated with smoking, drinking alcohol, diabetes history, and tumor size (Table 1).

**Table 1:** Clinicopathologic characteristics of study subjects.

Clinicopathologic characteristics	*n* = 21	Low (*n* = 9)	High (*n* = 12)	p-value
Age (years)				0.2563
< 65	11	6	5	
≥ 65	10	3	7	
Sex				0.8987
Male	12	5	7	
Female	9	4	5	
Smoking				0.0436*
No	11	7	4	
Yes	10	2	8	
Alcohol				0.0195**
No	8	6	2	
Yes	13	3	10	
Diabetes				0.0436*
No	11	7	4	
Yes	10	2	8	
Tumor size				0.0121*
≤ 4 cm	15	9	6	
> 4 cm	6	0	6	
Histology				0.0614
Grade 1	7	5	2	
Grade 2/3	14	4	10	
Metastasis				0.0562
No	9	6	3	
Yes	12	3	9	
Lymph node status				0.1946
Negative	8	2	6	
Positive	13	7	6	

### H3K18la is related to the severity and prognosis of pancreatic cancer

**Fig. 3:**
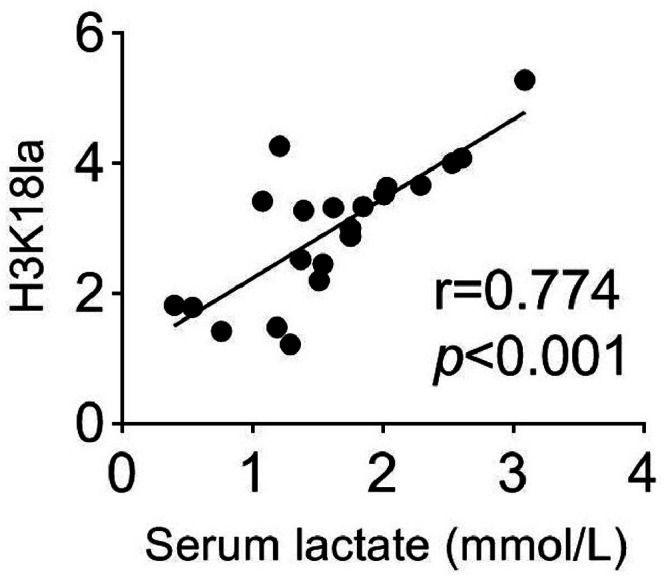
H3K18la levels were positively correlated with serum lactate in pancreatic cancer patients.

**Fig. 4:**
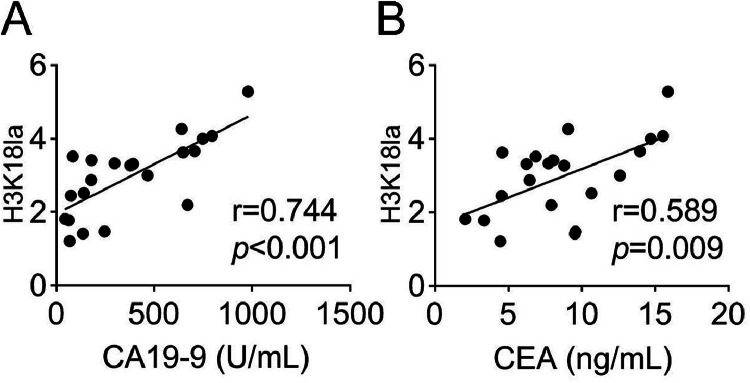
H3K18la levels were positively correlated with serum CA19–9 and CEA in pancreatic cancer patients.

**Fig. 5:**
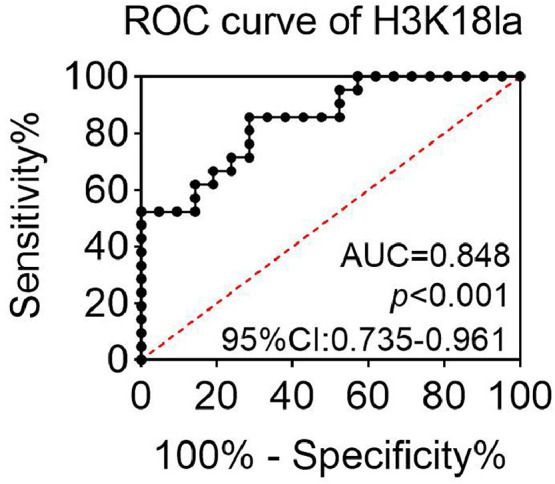
The Receiver Operating Characteristic (ROC) curve of H3K18la in pancreatic cancer.

There was a positive correlation between H3K18la and serum level of lactate (*r* = 0.774, *p* < 0.001) (Fig. 3). H3K18la also had a positive correlation with serum CA19–9 (*r* = 0.744, *p* < 0.001) and CEA (*r* = 0.589, *p* < 0.05) expression (Fig. 4A and B). In addition, the ROC analysis of H3K18la was performed, and the results suggested that the Area Under the ROC Curve (AUC) of H3K18la in serum was 0.848 (*p* < 0.001, 95 % CI 0.735‒0.961, Fig. 5), indicating the potential diagnostic value of H3K18la in pancreatic cancer.

## Discussion

In the current study, the authors first found that lactylation, a newfound protein post-translational modification, exists differentially in tumor tissues and adjacent tissues of pancreatic cancer. Moreover, H3K18la protein expression was also significantly elevated in tumor tissues compared with the normal controls. To our knowledge, this is the first clinical study exploring the relationship between H3K18la and pancreatic cancer. Importantly, the present study is the first to indicate that H3K18la correlates significantly with the severity and prognosis of patients with pancreatic cancer.

In the disease progression of pancreatic cancer, glycolysis provides the energy requirements for the growth of tumor cells, which leads to increased production of lactate.^15^ The accumulation of lactate not only reflects the high metabolic state of tumor cells but also may have an impact on surrounding tissues, including affecting normal cell function and microenvironment. For example, the accumulation of lactate may lead to local acidification, affecting cell survival and proliferation.^16^ Lactylation is a newly discovered post-translational protein modification in recent years. It is a kind of protein modification that covalently couples the lactoacyl group with protein lysine residue to promote gene regulation.^17^ A number of studies have shown that lactylation levels increase with the increase of intracellular lactate concentration in a dose-dependent manner.^11^^,^^18^, ^19^, ^20^, ^21^ Zhang et al. believe that lactylation is derived from lactate, and the use of γ-interferon combined with lipopolysaccharide or bacterial stimulation can increase the lactate produced by cells.^11^ Promoting glycolysis can increase intracellular lactate content and histone lactate levels. Pan et al. showed that the progression of Alzheimer's disease was promoted by a positive feedback loop of glycolysis-lactate-histone lactylation-glycolysis.^22^ Many studies have shown that lactate levels are reduced when the activity of lactate dehydrogenase, which catalyzes the oxidation of lactic acid to pyruvate, is inhibited.^11^^,^^23^ Yang et al. pointed out in more detail that inhibiting the expression of lactate dehydrogenase could significantly reduce histone lactylation in human renal carcinoma cells.^24^ Hence, the authors studied the histone lactylation in pancreatic cancer.

In this study, the authors first performed a small-sample-size preliminary experiment and found that lactylation is an all-protein post-translational modification that is present in tumor tissues and normal controls. H3K18la is a histone modification, which is the acetylated form on the 18th lysine of histone H3. This modification is often associated with increased transcriptional activity of genes, so it plays an important role in gene expression regulation.^25^ Abnormal expression or modification of H3K18la is associated with the occurrence and development of multiple tumor types.^26^^,^^27^ Then, the authors selected H3K18 as a modification site for detecting lactylation levels in pancreatic cancer patients, increased H3K18la protein levels were found in tumor tissues. Moreover, H3K18la displayed a positive correlation with serum lactate, CA19–9, and CEA. The ROC analysis also demonstrated that H3K18la may be an independent biomarker that reflects the severity and prognosis of pancreatic cancer.

In conclusion, H3K18la was up-regulated in pancreatic cancer and may function as a potential biomarker for diagnosis and prognosis of the disease. There are several limitations to this study. First, whether the regulation of pancreatic cancer process can be induced by H3K18la was not proved by the clinical findings. Second, this was a one-center, small-sample-size, historical cohort study, and several biases were inevitable. Further studies with larger sample sizes are needed to verify the present findings.

### Ethics approval and consent to participate

This study was performed in line with the principles of the ARRIVE guidelines. Approval was granted by the Ethics Committee of The Sixth People's Hospital of Chengdu (No.2020-L(thesis)−005). Informed consent was obtained from all individual participants included in the study.

### Consent for publication

Not applicable.

### Authors’ contributions

All authors participated in the design, interpretation of the studies, analysis of the data, and review of the manuscript. JP H and MS G drafted the work and revised it critically for important intellectual content; YQ L and YJ L were responsible for the acquisition, analysis, or interpretation of data for the work. All authors read and approved the final manuscript.

### Funding

The authors declare that no funds, grants, or other support were received during the preparation of this manuscript.

## Conflicts of interest

The authors declare no conflicts of interest.

## Data Availability

The datasets used and/or analyzed during the current study are available from the corresponding author upon reasonable request.
